# The changing demographics of the orthotist/prosthetist workforce in Australia: 2007, 2012 and 2019

**DOI:** 10.1186/s12960-021-00581-4

**Published:** 2021-03-17

**Authors:** Emily Ridgewell, Leigh Clarke, Sarah Anderson, Michael P. Dillon

**Affiliations:** 1 The Australian Orthotic Prosthetic Association, 2/1175 Toorak Road, Camberwell, VIC 3124 Australia; 2 Department of Physiotherapy, Podiatry, and Prosthetics and Orthotics; School of Allied Health, Human Services and Sport, La Trobe University, Melbourne, VIC 3086 Australia

**Keywords:** Workforce, Orthotist/prosthetist, Demographics, Allied health

## Abstract

**Background:**

Previous Australian workforce analyses revealed a small orthotist/prosthetist workforce with a low number of practitioners per 100,000 Australians. In recent years, initiatives were implemented to increase relative workforce size, including a government-led change in immigration policy to facilitate entry of experienced internationally trained orthotist/prosthetists into the Australian workforce. Given these changes, this project aimed to compare demographics of the orthotist/prosthetist workforce in Australia and each state/territory between 2007, 2012 and 2019.

**Methods:**

This quasi-experiment analysed data from the Australian Orthotic Prosthetic Association (AOPA) database of certified orthotist/prosthetists, to compare changes in the absolute number of practitioners and the number of practitioners per 100,000 population, as well as practitioner age, gender and service location (i.e., metropolitan, regional/remote) across three time points, with a breakdown by each Australian state and territory.

**Results:**

Between 2007 and 2019, the number of orthotist/prosthetists per 100,000 population increased 90%. Average age reduced significantly between 2007 (41.5 years) and 2019 (35 years) (*p* = 0.001). While the proportion of female practitioners increased significantly between 2007 (30%) and 2019 (49%), and between 2012 (38%) and 2019 (49%) (*p* < 0.05); only 22% of the female workforce is over 40 years of age. The proportion of practitioners servicing a regional/remote location did not change over time (range 13–14%).

**Conclusions:**

Between 2007 and 2019, the national orthotist/prosthetist workforce increased at a rate that exceeded Australia’s population growth, became younger, and more female. However, the number of practitioners per 100,000 population remains below international recommendations; particularly in states outside of Victoria and Tasmania, and in regional/remote areas. In addition, low numbers of mid-late career female practitioners suggest challenges to retention of this particular cohort. These data can help inform workforce initiatives to retain a younger and more female workforce, and improve access to orthotic/prosthetic services.

**Supplementary Information:**

The online version contains supplementary material available at 10.1186/s12960-021-00581-4.

## Background

Health workforce data is vital to meet the growing healthcare needs of our society [1, 2]. Allied health practitioners—including orthotists/prosthetists—represent approximately 20% of the healthcare workforce in Australia [3] and given the aging population, increased rates of chronic disease, and growing consumer expectations [1, 2, 4], will be increasingly relied upon to meet future healthcare needs.

Orthotist/prosthetists are tertiary qualified allied health practitioners trained to prescribe, design, fit and monitor orthoses (i.e., external braces or splints) and prostheses (i.e., artificial limbs) [5] across settings including primary care, disability and rehabilitation. Despite being one of the smallest allied health professions, representing an estimated 0.2% of the allied health practitioner workforce in Australia [6], orthotist/prosthetists provide highly specialised services to a broad range of clients across the lifespan—including people with limb loss, diabetic foot ulceration, stroke, cerebral palsy, scoliosis, post-polio syndrome, and lymphoedema, as well as management post-acute injury and surgery.

Small professions are particularly susceptible to changes in workforce supply and demand; as such, small professions are perhaps more reliant upon up-to-date and accurate workforce data to monitor changes and inform workforce planning. However, orthotist/prosthetists are one of several allied health professions omitted from federal workforce data sets [7, 8] partly due to the professions small size and the absence of federal regulation by the Australian Health Practitioner Regulation Agency (AHPRA). While the Australian census offers an alternative source of workforce data, census data falls rapidly out-of-date, includes data restrictions to ensure anonymity [9], and is likely affected by misclassification of related professionals who provide facial or breast prostheses, or who technicians who manufacture orthoses and prostheses.

For the orthotic/prosthetic workforce, the professional association database offers the most representative and reliable source of workforce data. The Australian Orthotic Prosthetic Association (AOPA) database of certified orthotist/prosthetists represent approximately 89% of practitioners in Australia [10], with certification restricted to practitioners that meet the entry-level requirements to provide patient care. The annual renewal process requires up-to-date personal details, and procedures are in place to identify and query aberrations.

Since data for the orthotist/prosthetist workforce were last published [11] the profession has experienced major changes to: technology, funding models and pathways to enter the profession. For example, advances in computer-aided design and improved access to centralised manufacturing and 3D printing technology has helped reduce the set-up costs for new services; in turn, this may have incentivised practitioners into private practice in under-serviced locations. Funding for orthoses and prostheses through the National Disability Insurance Scheme (NDIS) is now individualised in contrast to state-based block-funding models which has increased demand and further incentivised small, private practice growth. In response to the increased demand driven by the NDIS, and an identified immediate-[11] and projected medium-to-long term skills shortage, new pathways to enter the workforce were introduced. This included changes to immigration policy and a new bachelor-degree training program in Queensland.

Given such significant changes in the profession, it is timely to update the workforce data to inform continued workforce planning efforts. As such, the aim of this study was to compare demographics of the orthotist/prosthetist workforce in Australia and within each state/territory between 2007, 2012 and 2019. The rationale for comparison across these years included: earliest available workforce data for orthotist/prosthetists (i.e., 2007); pre-introduction of the NDIS trial sites which began in 2013 (i.e., 2012); and a follow-up year (i.e., 2019) which was 3-years post-NDIS roll-out, 2-years post-immigration policy change, and 1-year post-graduation of the first cohort from the new bachelor-degree course in Queensland, thus allowing sufficient lead time for these changes to impact the workforce. For example, 3-years post-introduction of the NDIS allows sufficient lead time for the creation of new jobs, 2-years post-immigration policy change allows for the lengthy timelines associated with assessment and migration of internationally qualified orthotist/prosthetists, whereas most graduates are employed within 12 months of graduation [12]. Consistent with the trends observed in a previous time-series [11] it was hypothesised that there would be a continued increase in practitioner prevalence, improved geographic dispersion of practitioners between states/territories, decreased mean age, increased proportion of female practitioners, and increased proportion of practitioners in regional/remote locations.

## Methods

Ethics approval was obtained from La Trobe University Human Research Ethics Committee.

### Study design

Quasi-experiment to compare workforce changes between three time points: 2007, 2012 and 2019.

### Data sources

Individual practitioner data were extracted from the AOPA database for the 2007, 2012 and 2019 certification years. Data from 2007 and 2012 were extracted as part of previous workforce analysis [11] and thus provide the first available workforce data for orthotist/prosthetists (i.e., 2007) and a snapshot of the workforce immediately prior to the introduction of the NDIS (i.e., 2012). The present study extends orthotist/prosthetist workforce data to 2019, thus capturing data that should reflect changes in the workforce in response to the roll-out of the NDIS (i.e., 2016), the immigration policy change (i.e., 2017) and graduation of first cohort from the new Bachelor-degree training program in Queensland (i.e., 2018).

Annual population data for the nation, each state/territory, and Remoteness Area, were obtained from the Australian Bureau of Statistics (ABS) [13, 14].

In 2007 and 2012, Remoteness Area was defined by the Australian Standard Geographical Classification (ASGC) 2006 [15, 16] and in 2019 the Australian Statistical Geography Standard (ASGS) 2016 [17] was used. Both classifications [15, 17] define Remoteness Area based on the Accessibility/Remoteness Index of Australia (ARIA) which measures the remoteness of a geographic location based on the physical road distance to the nearest urban centre in each of five size classes (i.e., major cities, inner regional, outer regional, remote and very remote) [17].

### Data extraction

De-identified practitioner data were exported from the AOPA database to a Microsoft (Redmond, WA, USA) Excel spreadsheet. Data were extracted prior to certification renewal (April) for both the 2007 and 2012 data, and immediately post-certification renewal (August) for the 2019 data.

Extracted data included: member number, membership category (i.e., certified, student, retired member, leave of absence), gender, date of birth, residential postcode; as well as workplace name, address and postcode. Only members certified to practice and who were employed in Australia were included. Therefore, student members, those employed overseas, on leave of absence (e.g., during a period of unemployment) or retired were excluded. Given the membership categories in the AOPA membership database, it was not possible to identify those who were clinically active as opposed to those members who work in academic, management or sales.

National, state/territory and Remoteness Area population data, captured on 30 June of each year [13, 14], were downloaded from the ABS.

### Data reduction

Age was determined from practitioner date of birth. For the 2007 and 2012 data, state/territory and Remoteness Area [15, 16] were determined using workplace postcodes. Where workplace postcodes from the 2019 data mapped to multiple Remoteness Areas [18], Remoteness Area was determined using the geocoder search function in ABS maps [19].

The five Remoteness Areas were collapsed into two service locations: ‘metropolitan’ included the Remoteness Area ‘major cities’; ‘regional/remote’ included ‘inner regional’, ‘outer regional’, ‘remote’ and ‘very remote’ [16]. Accordingly, all of Tasmania and the Northern Territory were considered ‘regional/remote’ across the time series.

Data were collated in Microsoft Excel (Redmond, WA, USA) and tabulated at a national, and state/territory-level for each year (i.e., 2007, 2012, 2019) such that it could be stratified to describe average practitioner age, and the proportion of practitioners according to gender, service location and 5-year age groups. Prevalence data (i.e., number of practitioners per 100,000 population) were calculated on a national level, for each state/territory and service location using the appropriate population data from the ABS [13, 14].

To explore changes in both practitioner age and gender, the proportion of male and female practitioners were reported according to 5-year age groups.

### Data analysis

Data were analysed on a national level and for each state/territory. Where data were not normally distributed both mean and medians were reported. Given that practitioner age was not normally distributed, changes in age over the time series were analysed using the Kruskal–Wallis *H* test with post-hoc comparisons performed using Dunn’s procedure with a Bonferroni correction for multiple comparisons. The Mann Whitney *U* test was used to analyse data from the Northern Territory between 2012 and 2019, given there were no AOPA members in the Northern Territory in 2007. The Chi-square test of homogeneity was used to determine whether change in the proportions of practitioners by gender and service location over time were due to chance. Post-hoc analysis involved pairwise comparisons using the *z*-test of two proportions with Bonferroni correction; or if fewer than five cases, the Fishers exact test. These analyses were conducted according to the techniques described by Lund and Lund [20, 21] using IBM SPSS Statistics 21 (IBM Corporation, Armonk, NY, USA).

## Results

### Practitioner number (number of orthotist/prosthetists)

**Fig. 1:**
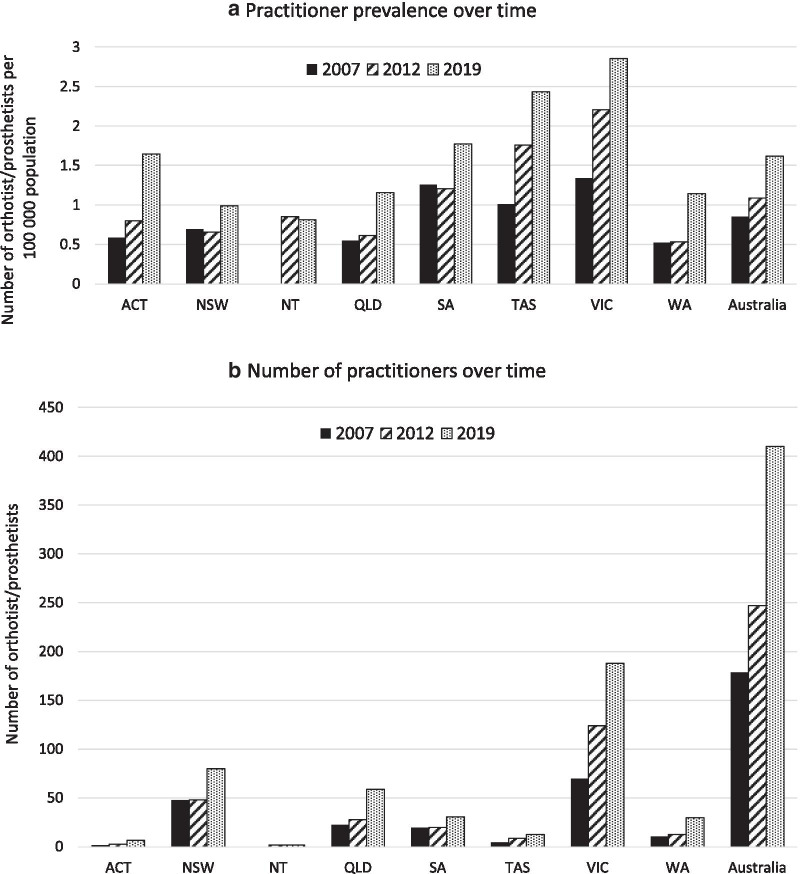
Prevalence and number of orthotist/prosthetists in Australia. **a** Practitioner prevalence and **b** absolute number of orthotist/prosthetists in Australia

Between 2007 and 2019, the number of practitioners in Australia increased by 130% with considerable variation between states/territories (Table 1; Fig. 1).

**Table 1:** Number of orthotist/prosthetists (and proportion of national workforce), practitioner prevalence, and population estimates [13] for each state/territory and national

	Number of practitioners (proportion of national workforce, %)			Practitioner prevalence (i.e., number of practitioners per 100,000 population)			Population estimates			
State	2007	2012	2019	2007	2012	2019	2007	2012		2019
ACT	2 (1)	3 (1)	7 (2)	0.59	0.80	1.64	342,644	376,539		426,704
NSW	48 (27)	48 (19)	80 (20)	0.70	0.66	0.99	6,834,156	7,304, 244		8,089,817
NT	0	2 (0.8)	2 (0.5)	0	0.85	0.81	213,748	235,915		245,929
QLD	23 (13)	28 (11)	59 (14)	0.55	0.61	1.16	4,111,018	4,568, 687		5,094,510
SA	20 (11)	20 (8)	31 (8)	1.26	1.21	1.77	1,570,619	1,656, 725		1,751,963
TAS	5 (3)	9 (4)	13 (3)	1.01	1.76	2.43	493,262	511,724		534,457
VIC	70 (39)	124 (50)	188 (46)	1.34	2.21	2.85	5,153,522	5,651, 091		6,596,039
WA	11 (6)	13 (5)	30 (7)	0.52	0.53	1.14	2,106,139	2,425, 507		2,621,509
Australia	179	247	410	0.85	1.09	1.62	20,827,622		22,733, 465	25,365,571

*ACT* Australian Capital Territory, *NSW* New South Wales, *NT* Northern Territory, *QLD* Queensland, *SA* South Australia, *TAS* Tasmania, *VIC* Victoria, *WA* Western Australia

### Practitioner prevalence (orthotist/prosthetists per 100,000 population)

Practitioner prevalence almost doubled over the time series with considerable variation between states/territories (Table 1; Fig. 1).

### Age

Over the time series, there was a significant change in median practitioner age of the national workforce (*χ*^2^(2) = 14.011, *p* = 0.001; Table 2; Additional file 1). Post-hoc comparisons showed a significant decrease in median practitioner age between 2007 and 2019 (Δ6.5 years, *p* = 0.001), but not between 2007 and 2012 (Δ4.5 years, *p* = 0.80) or between 2012 and 2019 (Δ2 years, *p* = 0.425).

**Table 2:** Median (and mean) orthotist/prosthetist age, proportion of workforce who were female, number and proportion who worked in a regional/remote location, and practitioner prevalence according to service location

State	Age				Gender				Service location								
	Median (mean) practitioner age			Post -hoc	Proportion (%) female			Post -hoc	Number (proportion; %) of practitioners in a regional/remote location			Practitioner prevalence i.e., number of practitioners per 100,000 population					
	2007	2012	2019		2007	2012	2019		2007	2012	2019	2007		2012		2019	
												Metro	Regional/ remote	Metro	Regional/ remote	Metro	Regional/ remote
ACT	43 (43)	27 (33)	32 (35)		0	0	29		0 (0)	0 (0)	0 (0)	0.58	0.00	0.80	0.00	1.64	0.00
NSW	49 (47)	48 (45)	34 (37)	^c^a, c	17	23	39	^c^a, b, c	2 (4)	2 (4)	7 (10)	0.91	0.11	0.85	0.11	1.18	0.40
NT^a,b^	–	37 (37)	52.5 (53)		–	0	0		–	2 (100^a^)	2 (100^a^)	–	–	N/A	0.85	N/A	0.81
QLD	44 (44)	34.5 (42)	33 (37)		22	18	42	^c^	4 (17)	3 (11)	9 (15)	0.75	0.25	0.88	0.17	1.52	0.50
SA	32 (38)	33 (36)	27 (33)		45	50	71		2 (10)	2 (10)	3 (10)	1.57	0.47	1.48	0.45	2.17	0.65
TAS^a^	34.5 (39)	45 (44)	45 (44)		40	33	62		5 (100^a^)	9 (100^a^)	13 (100^a^)	N/A	1.01	N/A	1.76	N/A	2.43
VIC	38 (39)	34 (37)	37 (39)		36	49	53	^c^a	10 (14)	21 (17)	24 (13)	1.54	0.80	2.38	1.59	3.18	1.66
WA	42 (42)	47 (46)	34.5 (38)		45	31	47		1 (9)	1 (8)	0 (0)	0.62	0.20	0.64	0.18	1.46	0.00
Australia	41.5 (42)	37 (39)	35 (38)	^c^a	30	38	49	^c^a, c	13	16	14	1.06	0.38	1.29	0.60	1.92	0.84

*Metro*.  metropolitan service location, *ACT* Australian Capital Territory, *NSW* New South Wales, *NT* Northern Territory, *QLD* Queensland, *SA* South Australia, *TAS* Tasmania, *VIC* Victoria, *WA* Western Australia

^a^All of the Northern Territory and Tasmania is considered regional/remote

^b^There were no AOPA members in the Northern Territory in 2007

^c^Significant main effect in the Kruskal Wallis *H* test or Pearson Chi Square test. Results of post-hoc testing: a = statistically significant difference between 2007 and 2019; b = statistically significant difference between 2007 and 2012; c = statistically significant difference between 2012 and 2019

State/territory analyses showed that only New South Wales had significant change in median practitioner age over the time series (*χ*^2^(2) = 17.209, *p* = 0.000; Table 2; Additional file 1). Post-hoc comparisons showed significant decrease in median practitioner age in New South Wales between 2007 and 2019 (Δ15 years, *p* = 0.001) and between 2012 and 2019 (Δ14 years, *p* = 0.007); but not between 2007 and 2012 (Δ1 year, *p* = 1.0). Median practitioner age did not change significantly over time in other states/territories.

### Gender

Over the time series, the proportion of female practitioners in the national workforce increased significantly (*χ*^2^(2) = 20.768, *p* = 0.000; Table 2; Additional file 1). Post-hoc comparisons showed a significant increase in the proportion of female practitioners between 2007 and 2019 (Δ19%, *p* < 0.05) and between 2012 and 2019 (Δ11%, *p* < 0.05); but not between 2007 and 2012.

There were significant increases in the proportion of female practitioners over the time series in New South Wales (*χ*^2^(2) = 8.172, *p* = 0.017), Victoria (*χ*^2^(2) = 6.255, *p* = 0.044) and Queensland (*χ*^2^(2) = 6.622, *p* = 0.036; Table 2). In New South Wales, post-hoc comparisons showed significant increases in the proportion of female practitioners between 2007 and 2019 (Δ22%, *p* < 0.05); between 2012 and 2019 (Δ16%, *p* < 0.05); and between 2007 and 2012 (Δ6%, *p* < 0.05). In Victoria, post-hoc comparisons showed significant increases in the proportion of female practitioners between 2007 and 2019 (Δ17%, *p* < 0.05); but not between 2007 and 2012, or between 2012 and 2019. Despite a significant main effect, in Queensland, post-hoc comparisons did not reveal any significant differences between years. The proportion of female practitioners did not change significantly over time in other states/territories (Table 2; Additional file 1).

### Age and gender

**Fig. 2:**
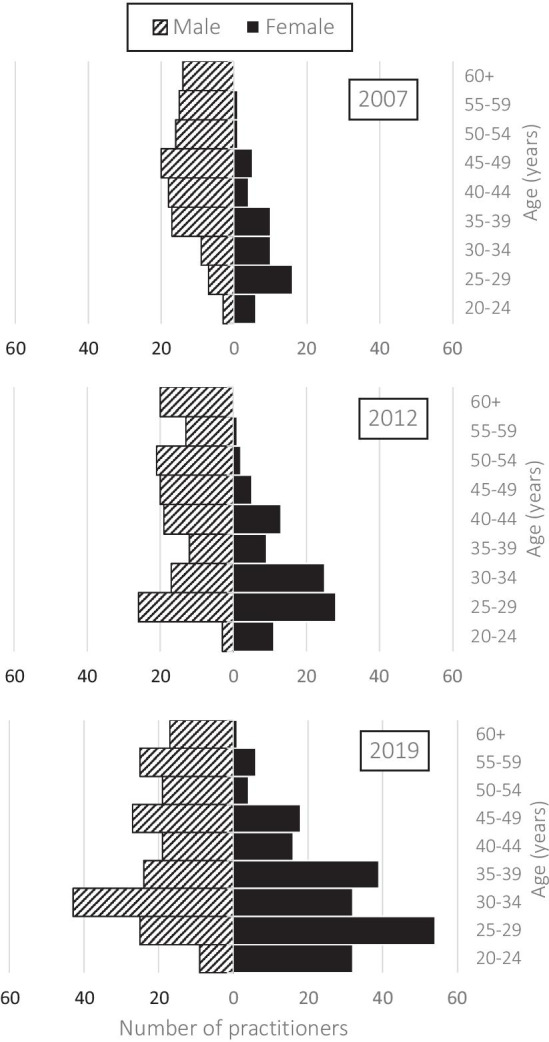
Age and gender of orthotist/prosthetists in Australia. Number of orthotist/prosthetists in Australia in 2019, stratified by gender and 5-year age groups

In 2019, 78% (*n* = 157) of all female practitioners were younger than 40 years, compared to half (49%; *n* = 101) of male practitioners (Fig. 2). In contrast, 5% (*n* = 11) of female practitioners were older than 50 years, compared to 30% (*n* = 61) of male practitioners.

These proportions are similar to those in 2007, with 80% (*n* = 43) of all female practitioners younger than 40 years compared to 34% (*n* = 42) of males; and 4% (*n* = 2) female practitioners older than 50 years compared to 36% (*n* = 45) of males.

### Service location

Over the time series, the proportion of the total workforce in regional/remote locations did not change (*χ*^2^(2) = 0.776, *p* = 0.679, Table 2, Additional file 1). Similarly, no states/territories demonstrated a significant change in the proportion of practitioners servicing a regional/remote location over time. However, between 2007 and 2019, practitioner prevalence in regional/remote locations more than doubled (Table 2).

## Discussion

Consistent with our hypothesis, the number of practitioners per 100,000 population increased 90% over the time series. While some of this growth will reflect the existing workforce taking up AOPA certification [10, 22], the remaining increase in the number of practitioners per 100,000 population is substantial, and consistent with real workforce growth. Despite this workforce growth, practitioner prevalence of 1.62 orthotist/prosthetists per 100,000 population is similar to current practitioner prevalence in the United Kingdom (1.64 per 100,000 population) [23] and the United States (1.7 per 100,000 population) (pers. comm., S. Fletcher—Director Professional Credentialling, American Board for Certification in Orthotics, Prosthetics and Pedorthics, 5th February 2021) but remains below recommendations for orthotists in Scotland (3.0 orthotists per 100,000 population) [24] and orthotist/prosthetists in the United States (2.4 per 100,000 population) [25, 26].

Contrary to our hypothesis, the geographic dispersion of practitioners did not improve over time, with nearly half of the workforce located in Victoria. Historically this concentration of practitioners in Victoria has been attributed to the location of, what was until recently, the only training program for orthotist/prosthetists in Australia. The prevalence of orthotist/prosthetists is not proportionate to the population in each state/territory; a challenge also reflected across the broader health workforce [4]. Some states had particularly low practitioner prevalence; for example, in 2019 both New South Wales and the Northern Territory had less than half the prevalence of Victoria, with fewer than one practitioner per 100,000 population. Encouragingly, New South Wales saw a 40% increase in practitioner prevalence between 2007 and 2019; however, this increase was less than half that observed in other states/territories. This likely points to barriers to workforce development in New South Wales, as evidenced by the recent focus of the Department of Health [27, 28] on workforce development strategies in this state. Lack of growth in practitioner prevalence in the Northern Territory is of concern given the high incidence of diabetes—particularly among Indigenous Australians [29]—which is a common precursor to foot ulceration and amputation; both of which require the specialist services of orthotist/prosthetists. Low practitioner prevalence indicates a workforce that is likely stretched thin, especially in states outside of Victoria and Tasmania (refer to Table 1). This is likely to have a negative impact on staff (e.g., stress, burnout, mental health) which in turn impacts quality of care (e.g., patient safety), as noted in other health professions [30–32].

Consistent with our hypothesis, the national workforce became younger which was in contrast to the ageing workforce seen in the majority of regulated allied health professions [33–42]. It is not likely that a younger workforce is attributable to growth in graduate numbers, given there has not been a substantial increase in graduate numbers per annum over the time series from the Victorian training program, (average 34 practitioners per annum) (pers. comm., M. Dillon—Head of Department Physiotherapy, Podiatry, Prosthetics and Orthotics, La Trobe University, 5th May 2020) with a total of ten practitioners graduated from the new Queensland training program across the 2 years of 2018 and 2019 (pers. comm., B. Delaney—Program Coordinator of Prosthetics and Orthotics, School of Health and Behavioural Sciences, University of the Sunshine Coast, 27th January 2021). This small increase in graduate numbers over 2018–2019 is likely offset by the older age (average 42 years) of the small number of practitioners who entered the workforce by the immigration pathway and have remained in the workforce since that time; approximately 2–3 practitioners per annum. Our results suggest that the average age of practitioners has reduced due to the attrition of mid-career practitioners. For example, in 2019, the number of male practitioners reduced sharply at age 35 and the number of females reduced sharply in each of the 30-, 40-, and 50-year age groups (Fig. 2).

Consistent with our hypothesis, the orthotist/prosthetist workforce became more female; a trend occurring in other health professions in Australia [34, 35, 37, 38, 42, 43] and globally [44]. Sixty percent of all graduates are female (SD 4.7) (pers. comm., M. Dillon—Head of Department Physiotherapy, Podiatry, Prosthetics and Orthotics, La Trobe University, 5th May 2020; pers. comm., B. Delaney—Program Coordinator of Prosthetics and Orthotics, School of Health and Behavioural Sciences, University of the Sunshine Coast, 27th January 2021) a trend that has remained relatively stable over the time series. While more females are entering the profession, older male practitioners are retiring; a trend likely to continue given that over the next 15 years, 29% of male practitioners will reach retirement age (i.e., age 65) compared to only 5% of female practitioners (Fig. 2).

While the proportion of young female practitioners is growing, the proportion of female mid-late career practitioners shrinks rapidly around the age of 40, with only 22% (*n* = 45) of female practitioners over the age of 40, compared to 49% (*n* = 107) of males (Fig. 2). High attrition of mid-to-late career female practitioners may reflect a lack of available part-time roles to support females in the workforce, given females generally work fewer hours than their male counterparts [33–43, 45] as evidenced by only 4–5% of fathers working part-time compared to 40% of mothers [46]. Loss of mid-late career female practitioners is likely to substantially reduce future workforce capacity, especially given the large cohort of young females currently entering the profession.

Also of concern is the impact of poor age-gender diversity of the orthotist/prosthetist workforce, and the impact this likely has on the quality of care. Lack of diversity in the healthcare workforce has been linked to health care disparities [47–51], thus the absence of mid-late career female orthotist/prosthetists who share similar lived experiences with older female patients, will limit the capacity of the workforce to provide truly nuanced, patient-centred care. In addition, fewer mid-to-late career female practitioners will mean fewer women represented in leadership roles, whereby they can advocate for, and inform changes [52] to remove system, policy and culture barriers, to help retain future female practitioners and restore gender balance in the mid-late practitioner cohort.

In contrast to our hypothesis, the proportion of the national workforce in regional/remote locations did not change over time (Table 2). In keeping with other health professions [34–43] approximately 14% of the workforce was located in regional/remote locations of Australia, including all of Tasmania and the Northern Territory (Table 2). By contrast, approximately 27% of the population is located in regional/remote locations of Australia [14].

While the proportion of the workforce in regional/remote areas was unchanged, practitioner prevalence more than doubled, which should translate into improved access to services. Despite this increase in practitioner prevalence, these areas are under-serviced given prevalence remains less than half that of metropolitan areas. Acknowledging that many metropolitan-based service providers do provide out-reach services into regional/remote areas, it remains that access to health workers is generally poorer in regional/remote locations compared to metropolitan centers [4]. Thus, for people accessing orthotic/prosthetic care, this likely means an increased wait time for available appointments, travel burden and delays (e.g., travelling several hours to an appointment) and poor access to practitioners with sub-speciality expertise (e.g., upper-limb prosthetics), which will negatively impact health outcomes and hinder a person’s ability to participate in society (e.g., ability to work). Given that people living in remote/regional locations are already at risk for worse health outcomes [53], targeted workforce planning initiatives that address barriers to recruitment and retention—such as remuneration, professional demands, education opportunities and lifestyle concerns [4]—of orthotist/prosthetists in regional/remote areas should remain a priority.

### Future research

These results indicate challenges in maintaining practitioners in the national workforce; in particular females over the age of 40 years. Identifying factors that influence workforce attrition such as lack of career progression [54] or poor workplace flexibility [55], and workforce barriers in locations with continued low practitioner prevalence, would allow the development of targeted initiatives. Future studies should consider the impact of the immigration pathway and a new training program on workforce distribution. Similarly, waiting-time or job vacancy data could be used as an indication of workforce under- or over- supply. The use of stock and flow models could be considered as an alternative approach to estimating new entrants to the workforce, and those leaving the profession.

### Limitations

Given AOPA certification is required for most, but not all funded clinical care, approximately 11% of the 2019 workforce are not in the AOPA database [10]. The number of practitioners in the database has increased over time (approximately 57% [22] in 2011; approximately 89% [10] in 2016), so some workforce growth reflects the existing workforce taking up AOPA certification. Practitioners work across a range of areas (e.g., academia, management and sales), and practitioners taking up a leave-of-absence may choose to re-certify during a membership year, so full-time equivalent positions dedicated to patient care are not known. While these issues affect the accuracy of the absolute number of practitioners, all time points are equally affected, and therefore, comparisons over time are fair.

While comparing Australian practitioner prevalence with international counterparts provides context for better understanding workforce shortage, county-specific certification requirements will influence the confidence with which we interpret these comparisons. For example, similar to Australian requirements, certification as a prosthetist/orthotist is not legislated in some parts of the United States, thus prevalence calculations in both Australia and the United States may be slightly underestimated compared to the United Kingdom, where certification is required to practice.

Extracting member data immediately pre- certification renewal, in both 2007 and 2012, will result in a small overestimation of practitioner numbers; estimated to be in the order of 1% given a small number of practitioners who did not renew their certification in 2018–2019. Extracting member data immediately post- certification renewal, as occurred in 2019, is planned for future data collection.

The use of two geographical classification systems [15, 17] in this time series reflects the regular updates to the standard framework for statistical geography by Government agencies. Both classifications determine Remoteness Areas using the ARIA, so it is likely to have little impact on Remoteness Area classification across the time-series.

Despite a significant main effect of practitioner gender in Queensland, post-hoc comparisons did not reveal a significant difference between years. The lack of statistical significance likely reflects large variability and few data points, so much so that large proportionate changes are not reflected in results of the inferential analysis. Readers should carefully consider the findings of the inferential analysis in light of the descriptive data.

## Conclusion

The national orthotist/prosthetist workforce more than doubled between 2007 and 2019. The number of practitioners per 100,000 population grew substantially, indicating growth in the workforce that outpaced population growth. While the workforce is becoming younger and more female, the low number of mid-career female practitioners suggests difficulties retaining this cohort which is of concern given that females in senior roles—particularly those in leadership roles—are best placed to create a workplace that better meets the needs of a more femanised workforce. There are also challenges in providing greater access to services given the narrow geographic dispersion of practitioners across states/territories and regional/remote areas. These data allow workforce initiatives to be tailored meet state/territory-specific needs and help improve access to orthotic/prosthetic services for all Australians.

## Supplementary Information

**Additional file 1**. Supplementary material to “The changing demographics of the orthotist/prosthetist workforce in Australia: 2007, 2012 and 2019”. Full results of the inferential analysis. This additional material reports the complete findings of the inferential statistics.

## Data Availability

The data that support the findings of this study are available from the Australian Orthotic Prosthetic Association but restrictions apply to the availability of these data, given concerns regarding data anonymity.

## Abbreviations

AOPA: Australian Orthotic Prosthetic Association
ABS: Australian Bureau of Statistics
NDIS: National Disability Insurance Scheme
