# Supplementary material for: The changing demographics of the orthotist/prosthetist workforce in Australia: 2007, 2012 and 2019
Source: Hum Resour Health. 2021 Mar 17;19:34. doi: 10.1186/s12960-021-00581-4 (PMC7968165; doi:10.1186/s12960-021-00581-4)
Supplement: Supplementary file 1 — Additional file 1. Supplementary material to “The changing demographics of the orthotist/prosthetist workforce in Australia: 2007, 2012 and 2019”. Full results of the inferential analysis. This additional material reports the complete findings of the inferential statistics. [file 12960_2021_581_MOESM1_ESM.docx]

# Supplementary material to “The changing demographics of the orthotist/prosthetist workforce in Australia: 2007, 2012 and 2019“.

Table 1. Full results of the inferential analysis.

| A Kruskal-Wallis H test was conducted to determine if there were differences in practitioner age between years. Pairwise comparisons were performed using Dunn's (1964) procedure with a Bonferroni correction for multiple comparisons. Adjusted p values are presented. | | | | |
| --- | --- | --- | --- | --- |
| **Practitioner age** | | | | |
| *State* | *H* | *DF* | *p* | *Post hoc* |
| ACT | 0.752 | 2 | 0.687 |  |
| QLD | 4.867 | 2 | 0.088 |  |
| **NSW** | **17.209** | **2** | **0.000** | **Significant difference between 2007 and 2019 (p=0.001); 2012 and 2019 (p= 0.007) but not 2007 and 2012 (p=1.0)** |
| NT# | 4 |  | 0.333 |  |
| SA | 2.059 | 2 | 0.357 |  |
| TAS | 0.369 | 2 | 0.831 |  |
| VIC | 4.466 | 2 | 0.107 |  |
| WA | 3.449 | 2 | 0.178 |  |
| **Australia** | **14.011** | **2** | **0.001** | **Significant difference between 2007 and 2019 (p=0.001) but not 2007 and 2012 (p= 0.80) or between 2012 and 2019 (p=0.425)** |
| # Mann Whitney U test was used because there was only 2 years of data. | | | | |
|  |  |  |  |  |
| Pearson chi square test of homogeneity is used to determine if a difference exists between the binomial proportions of three or more independent groups on a dichotomous dependent variable. Post hoc analysis involved pairwise comparisons using the z-test of two proportions with a Bonferroni correction. | | | | |
| **Gender** | | | | |
| *State* | *Test statistic* | *DF* | *p* | *Post hoc* |
| ACT $ |  | | | |
| **QLD** | **6.622** | **2** | **0.036** | **No post hoc differences** |
| **NSW** | **8.172** | **2** | **0.017** | **Significant difference between 2007 and 2012; 2012 and 2019; and 2007 and 2019 (p<0.05)** |
| NT $ |  | | | |
| SA | 4.044 | 2 | 0.132 |  |
| TAS | ^Fishers exact test 0.205 | | 1.00 |  |
| **VIC** | **6.255** | **2** | **0.044** | **Significant difference between 2007 and 2019 (p<0.05)** |
| WA | ^ Fishers exact 1.131 | | 0.540 |  |
| **Australia** | **20.768** | **2** | **0.00** | **Significant differences between 2007 and 2019, and 2012 and 2019 (p<0.05)** |
| **Service location (proportion rural/remote)** | | | | |
| ACT~ |  | | | |
| QLD | ^Fisher exact test: 0.554 | | 0.822 |  |
| NSW | ^ Fishers exact test: 1.282 | | 0.561 |  |
| NT~ |  | | | |
| SA | 0.002 | 2 | 0.999 |  |
| TAS~ |  | | | |
| VIC | 1.055 | 2 | 0.59 |  |
| WA | ^ Fishers exact test 2.996 | | 0.21 |  |
| Australia | 0.776 | 2 | 0.679 |  |
| ^ Fishers exact test was used because some cells had counts less than 5. $ Sample size less than 5; unable to compute Fishers exact test because ‘cases with zero, negative, or missing values for the weighting variable are excluded from analysis.’ Two years have zero cases so will be excluded. ~ all of NT and TAS are considered rural/remote. | | | | |

Table 2. Number (and proportion) of practitioners stratified by gender and age

|  |  | Number (proportion) of practitioners stratified by gender and age | | | | | | | | | | |
| --- | --- | --- | --- | --- | --- | --- | --- | --- | --- | --- | --- | --- |
|  |  | Unknown | 20-24 | 25-29 | 30-34 | 35-39 | 40-44 | 45-49 | 50-54 | 55-59 | 60-64 | 65+ |
| 2007 | Female | 1 (2) | 6 (11) | 16 (30) | 10 (19) | 10 (19) | 4 (7) | 5 (9) | 1 (2) | 1 (2) |  |  |
|  | Male | 6 (5) | 3 (2) | 7 (6) | 9 (7) | 17 (14) | 18 (14) | 20 (16) | 16 (13) | 15 (12) | 9 (7) | 5 (4) |
| 2012 | Female |  | 11 (12) | 28 (30) | 25 (27) | 9 (10) | 13 (14) | 5 (5) | 2 (2) | 1 (1) |  |  |
|  | Male | 2 (1) | 3 (2) | 26 (17) | 17 (11) | 12 (8) | 19 (12) | 20 (13) | 21 (14) | 13 (8) | 14 (9) | 6 (4) |
| 2019 | Female |  | 32 (16) | 54 (27) | 32 (16) | 39 (19) | 16 (8) | 18 (9) | 4 (2) | 6 (3) | 1 (<1) |  |
|  | Male |  | 9 (4) | 25 (12) | 43 (21) | 24 (12) | 19 (9) | 27 (13) | 19 (9) | 25 (12) | 9 (4) | 8 (4) |
